# Bilateral Lung Transplantation for Lymphangioleiomyomatosis With Secondary Pulmonary Hemosiderosis: A Case Report

**DOI:** 10.1155/crip/9989977

**Published:** 2026-04-07

**Authors:** Fabio Varon-Vega, Eduardo Tuta-Quintero, David Mendoza, Luis Jaime Tellez, Camilo Rodriguez, Jacqueline Mugnier, María Camila Martínez-Ayala

**Affiliations:** ^1^ Critical Care Service, Fundación Cardioinfantil-Instituto de Cardiología, Bogotá, Colombia, cardioinfantil.org; ^2^ Critical Care and Lung Transplantation Service, Fundación Neumológica Colombiana, Bogotá, Colombia; ^3^ School of Medicine, Universidad de La Sabana, Chía, Colombia, unisabana.edu.co; ^4^ Department of Pathology, Fundación Cardioinfantil-Instituto de Cardiología, Bogotá, Colombia, cardioinfantil.org

**Keywords:** critical care, extracorporeal membrane oxygenation, lung transplantation, lymphangioleiomyomatosis, pulmonary hemosiderosis, respiratory insufficiency

## Abstract

**Background:**

Pulmonary lymphangioleiomyomatosis (LAM) is a rare and progressive disease characterized by abnormal proliferation of smooth muscle–like cells leading to diffuse cystic destruction of the lung parenchyma and respiratory compromise, predominantly affecting young women. Idiopathic pulmonary hemosiderosis (IPH), a rare disorder characterized by recurrent diffuse alveolar hemorrhage and iron deposition in the lungs, may present with nonspecific respiratory symptoms and radiologic findings that can overlap with other diffuse lung diseases, potentially leading to diagnostic confusion. Such overlap may delay accurate diagnosis and appropriate management.

**Case Presentation:**

We present the case of a 29‐year‐old woman with a prior diagnosis of IPH who was listed for lung transplantation. She presented with severe acute respiratory decompensation and required urgent bilateral lung transplantation. Due to intraoperative hemodynamic instability, she underwent the procedure under venoarterial extracorporeal membrane oxygenation (VA‐ECMO) support. Histopathological analysis of the explanted lungs revealed pulmonary LAM as the actual underlying condition, not IPH.

**Conclusion:**

This case highlights the diagnostic complexity of rare pulmonary diseases and the potential for misdiagnosis, particularly in advanced stages. It underscores the importance of considering LAM in the differential diagnosis of diffuse lung diseases in young women and demonstrates the critical role of lung transplantation and early multidisciplinary intervention in managing end‐stage respiratory failure due to rare etiologies.

## 1. Introduction

Idiopathic pulmonary hemosiderosis (IPH) is a rare cause of diffuse alveolar hemorrhage, clinically characterized by recurrent hemoptysis and, in the long term, respiratory failure due to chronic alveolar damage [1, 2]. Diagnosis is often delayed and requires histological confirmation [2]. Its pathogenesis remains unclear, and treatment is not well defined, although corticosteroids and immunosuppressive therapies are commonly used [2, 3]. In advanced stages, lung transplantation has been considered a therapeutic option, although there is a risk of disease recurrence in the graft [1–4].

Pulmonary lymphangioleiomyomatosis (LAM) is also a rare and progressive disease, estimated to affect 3.4–7.8 per million women, and is characterized by abnormal proliferation of smooth muscle–like cells infiltrating the lungs, leading to diffuse cystic parenchymal destruction and progressive respiratory impairment [5–8]. Clinically, patients may present with dyspnea, recurrent pneumothorax, chylous effusions, or progressive respiratory failure. Diagnosis is based on characteristic findings on high‐resolution computed tomography (HRCT), frequently supported by elevated vascular endothelial growth factor‐D (VEGF‐D) levels or histopathological confirmation. The disease is associated with dysregulation of the mTOR pathway, and treatment with mTOR inhibitors such as sirolimus has been shown to stabilize lung function. Lung transplantation remains an option in advanced disease, although recurrence of LAM in the transplanted lung has been reported. Due to overlapping clinical or radiologic features, LAM can occasionally mimic IPH or other diffuse lung diseases in young women, creating diagnostic challenges [8, 9].

We report the case of a young woman with a prior diagnosis of IPH who presented in critical condition with acute‐on‐chronic respiratory failure. She underwent urgent bilateral lung transplantation under extracorporeal membrane oxygenation (ECMO) support. Histopathological examination of the explanted lungs revealed LAM, illustrating the diagnostic challenges and the importance of considering this entity in the differential diagnosis of rare diffuse lung diseases.

## 2. Case Report

**Figure 1:**
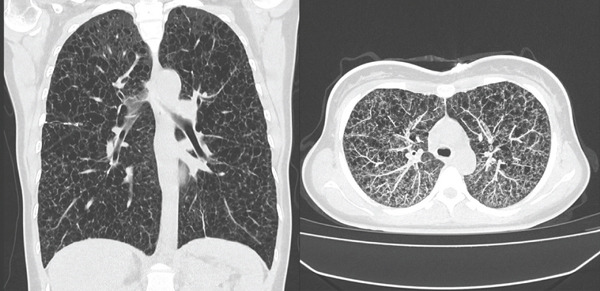
Chest computed tomography.

A 29‐year‐old woman with a prior diagnosis of IPH, established approximately 10 years prior and listed for lung transplantation, was transferred by ambulance from a chronic care facility to the emergency department of Fundación Cardioinfantil–LaCardio, where she was evaluated by the Critical Care and Lung Transplantation Service teams in collaboration with Fundación Neumológica Colombiana, Bogotá, Colombia, due to progressive dyspnea over 3 days, accompanied by severe desaturation down to 30% and oppressive retrosternal chest pain. Chest computed tomography showed bilateral increased lung volume secondary to multiple thin‐walled cystic lesions with diffuse distribution in both lung fields, associated with areas of increased density relative to normal lung parenchyma (Figure 1).

Upon arrival, she was alert, with acceptable vital signs while on high‐flow nasal cannula (HFNC) oxygen support, experiencing severe dyspnea despite morphine treatment. She required progressively increasing oxygen flow and inspired oxygen fraction (FiO_2_), remaining severely functionally limited. In the context of acute‐on‐chronic respiratory failure and clinical deterioration, the transplant team was notified of compatible donor organs. Following interdisciplinary evaluation, she was deemed suitable for bilateral lung transplantation under emergency status. All procedures related to organ procurement and transplantation were conducted in accordance with Colombian legal regulations and institutional transplantation protocols.

The donor lungs appeared macroscopically normal, with no adhesions or nodular lesions, and passed the deflation test satisfactorily. However, retrograde perfusion revealed blood‐tinged reflux, prompting lavage with 3 L of Perfadex. The implantation proceeded without major technical difficulties, though numerous hilar lymph nodes were noted, which were within expected macroscopic size limits and showed no gross signs of pathological enlargement.

During the left pneumonectomy, severe intraparenchymal bleeding occurred, associated with spontaneous hematomas, resulting in hemodynamic instability and intraoperative cardiac arrest. Cardiopulmonary resuscitation was initiated, including cardiac massage, epinephrine administration, and peripheral venoarterial extracorporeal membrane oxygenation (VA‐ECMO) support, with venous cannulation in the right femoral vein and arterial cannulation in the left femoral artery via a vascular graft. Intraoperative transfusion was complicated by episodes of ventricular arrhythmias requiring defibrillation. In the immediate postoperative period, the patient developed vasoplegic shock, likely secondary to myocardial stunning, requiring vasopressor and inotropic support with norepinephrine, vasopressin, and milrinone.

Due to persistent bleeding in the first hours, a surgical reintervention was performed, revealing 3 L of clots in the pleural cavity without a clear hemorrhagic source, followed by pleural packing. Subsequently, the patient showed progressive improvement, with hemodynamic stabilization, bleeding control, resolution of hyperlactatemia, and adequate pulmonary expansion on follow‐up imaging. Following a favorable course, VA‐ECMO was successfully weaned on postoperative Day 22.

**Figure 2:**
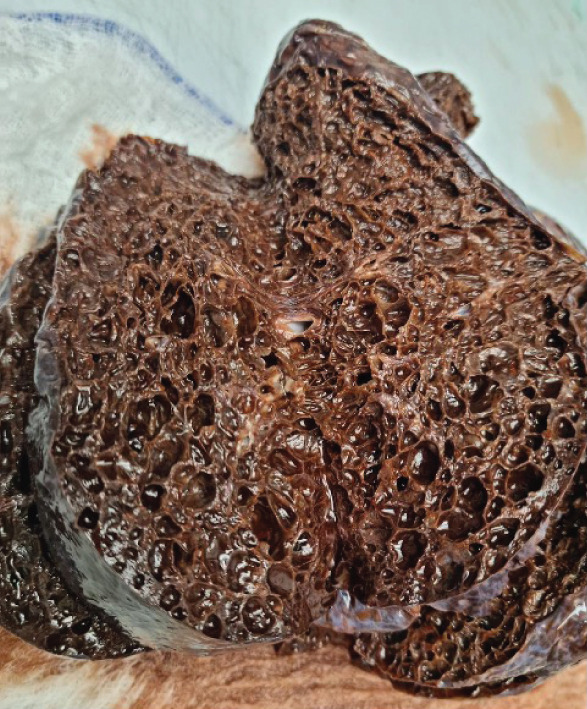
Explanted lung.

Gross examination of the explanted lungs revealed markedly abnormal architecture (Figure 2). The external surface was smooth with a violaceous appearance. The pulmonary parenchyma showed multiple dilated cystic spaces ranging from 0.2 to 2 cm, without evidence of solid lesions. Numerous hilar lymph nodes were noted. Sectioning revealed copious hemorrhagic effusion and widespread cystic changes.

Histopathological analysis confirmed the diagnosis of LAM (Figure 3). There was widespread involvement of the lung parenchyma with abnormal smooth muscle proliferation leading to cystic destruction. Additionally, there was extensive alveolar hemorrhage and marked pulmonary hemosiderosis, consistent with the patient’s pre‐existing diagnosis of IPH, now interpreted as secondary to LAM. Immunohistochemical staining demonstrated strong cytoplasmic positivity for smooth muscle actin and HMB‐45, along with nuclear expression of estrogen and progesterone receptors in the neoplastic cells, supporting the diagnosis of LAM. Melan‐A staining was negative. The combination of characteristic spindle‐shaped smooth muscle–like cells and this immunohistochemical profile confirmed the diagnosis of pulmonary LAM.

Figure 3Histopathological findings of the explanted lung. (a) Hematoxylin–eosin stain, 40× objective, showing spindle‐shaped neoplastic cells with oval nuclei consistent with modified smooth muscle cells (LAM cells). (b) Hematoxylin–eosin stain, 10× objective, showing architectural distortion of lung parenchyma with abundant intra‐alveolar hemosiderin‐laden macrophages and thickening of alveolar septa. (c) Smooth muscle actin (SMA) immunohistochemistry, 10× objective, demonstrating cytoplasmic positivity in neoplastic cells. (d) HMB‐45 immunohistochemistry, 10× objective, showing cytoplasmic positivity in neoplastic cells. (e) Estrogen receptor immunohistochemistry, 10× objective, demonstrating nuclear positivity in neoplastic cells. (f) Progesterone receptor immunohistochemistry, 10× objective, demonstrating nuclear positivity in neoplastic cells.

**Figure:**
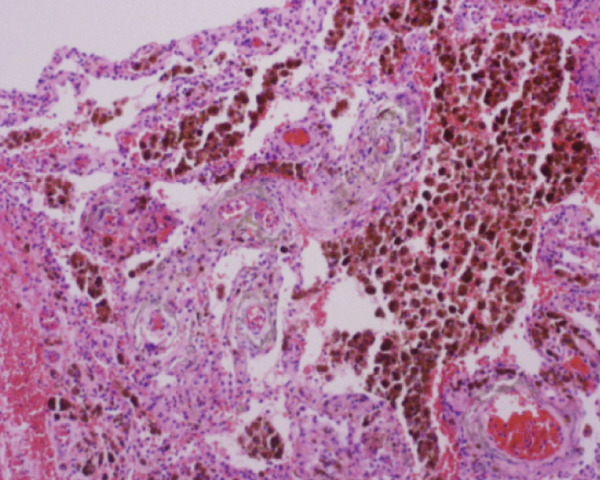
(a)

**Figure:**
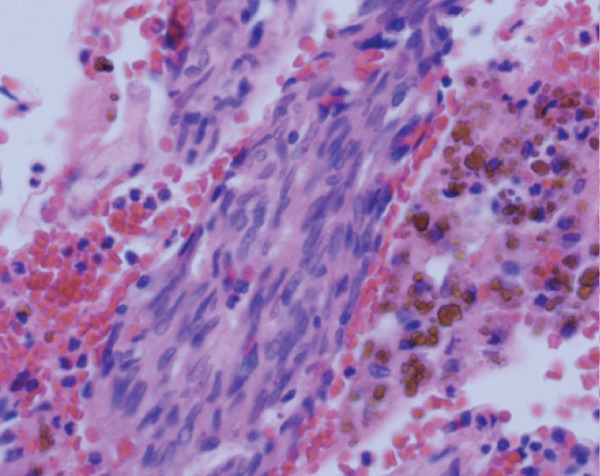
(b)

**Figure:**
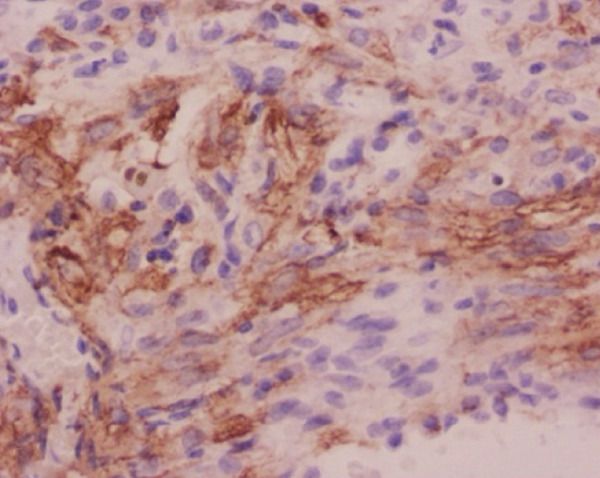
(c)

**Figure:**
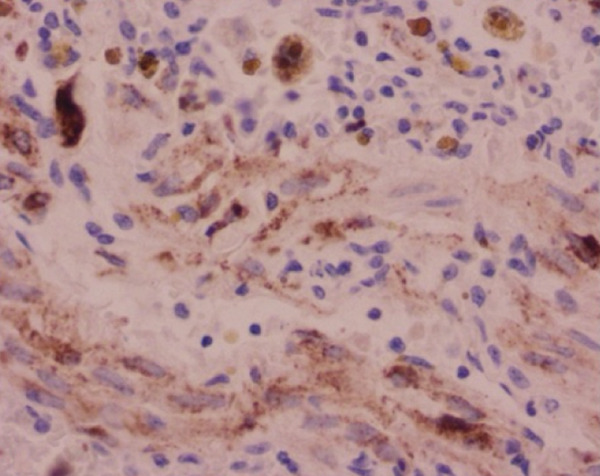
(d)

**Figure:**
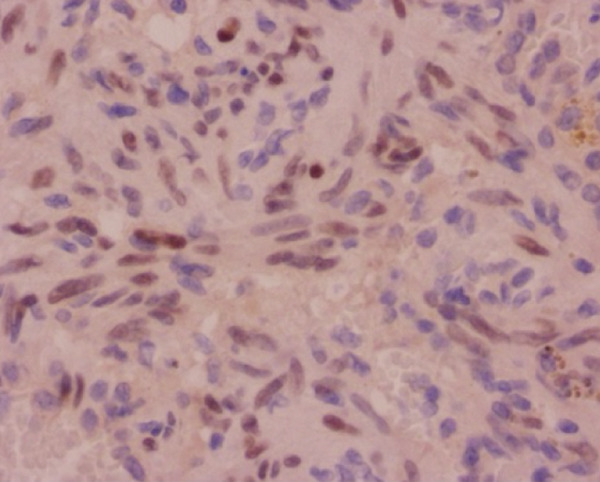
(e)

**Figure:**
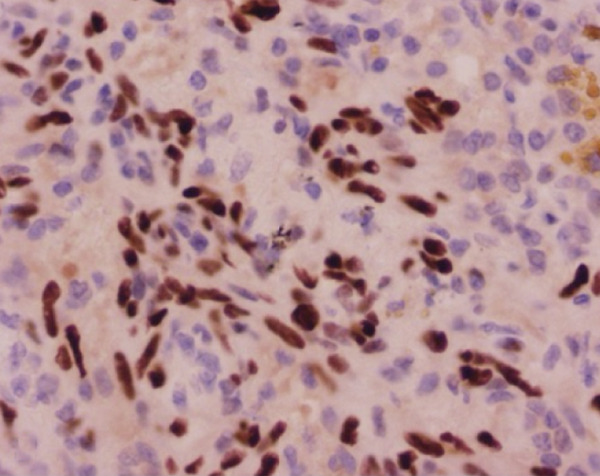
(f)

## 3. Discussion

This case presents a young woman with a history of IPH who required urgent bilateral lung transplantation due to acute‐on‐chronic respiratory failure, supported by ECMO. However, postoperative histopathology revealed LAM, a rare, progressive, and often underdiagnosed condition that predominantly affects women of reproductive age [6, 7]. The diagnosis was ultimately confirmed by histopathology, showing proliferation of spindle‐shaped smooth muscle–like cells and a characteristic immunohistochemical profile with positivity for smooth muscle actin, HMB‐45, and hormone receptors, which is diagnostic of LAM.

LAM is a low‐grade metastatic neoplasm infiltrating the pulmonary parenchyma, causing diffuse cystic destruction, airflow obstruction, and lymphatic complications such as chylothorax, ascites, and recurrent spontaneous pneumothorax [8, 10–12]. Clinically, it presents with a range of symptoms that significantly impact quality of life, including dyspnea, dry cough, and pneumothorax episodes [7]. In this case, the patient had progressive dyspnea, alveolar hemorrhage, and severe hypoxemia, initially interpreted as IPH. However, intraoperative findings of intraparenchymal hematomas and tissue fragility suggested an alternative pathology consistent with LAM.

LAM diagnosis requires a high index of clinical suspicion and relies on HRCT findings and histopathological confirmation, particularly in young women with cystic lung disease [10–12]. In our case, the absence of a pretransplant diagnosis emphasizes the difficulty in identifying this disease at advanced stages or with atypical presentations, especially when dominated by hemorrhagic manifestations. LAM can mimic other interstitial lung diseases due to clinical variability and the presence of obstructive lung function, which often leads to initial misdiagnosis as asthma or COPD, with up to one‐third of cases initially misdiagnosed [7, 12, 13].

During surgery, the patient experienced severe complications, including uncontrolled bleeding, cardiac arrest, and hemodynamic instability, requiring VA‐ECMO support. This reflects the typical complications in patients with advanced LAM, where structural alterations of lung tissue, lymphatic disruption, and abnormal vascularization significantly increase surgical risk [5, 8]. Despite these challenges, the patient achieved clinical stabilization, successful ECMO weaning by Day 22, and progressive functional recovery, underscoring the importance of intensive multidisciplinary care and advanced perioperative support.

Although LAM usually follows a slow course, it can progress to end‐stage respiratory failure. Median transplant‐free survival exceeds 20 years from diagnosis, with 10‐year survival rates above 85% in several international cohorts [14, 15]. However, progressive functional decline and complications, such as recurrent pneumothorax, hemorrhage, or chylothorax, may impair quality of life and justify lung transplantation in advanced stages [5, 8].

Adult‐onset IPH is rare, accounting for approximately 20% of reported cases, with most diagnoses occurring between the third and fourth decades of life [16–18]. This distribution underscores that adult presentations are uncommon and may display clinical features that differ from those seen in pediatric patients [16–18]. Published case reports further highlight the rarity of this condition in adults and stress the importance of thoroughly excluding alternative causes of diffuse alveolar hemorrhage before confirming the diagnosis.

## 4. Conclusion

This case shows the diagnostic challenges of rare pulmonary diseases such as LAM, which can mimic other pathologies. It emphasizes the importance of accurate differential diagnosis and the role of lung transplantation within a timely multidisciplinary approach in advanced disease stages, not only as a therapeutic intervention but also as a diagnostic tool in end‐stage lung disease of unclear etiology. Early recognition of LAM could allow timely referral for transplant evaluation and consideration of targeted therapies.

## Author Contributions

All authors contributed substantially to the conception, design, drafting, and revision of the manuscript.

## Funding

No funding was received for this manuscript.

## Disclosure

All authors read and approved the final version of the manuscript.

## Ethics Statement

The authors have nothing to report.

## Consent

Written informed consent was obtained from the patient for inclusion in this study and for the publication of anonymized clinical data, this case report, and any accompanying images.

## Conflicts of Interest

The authors declare no conflicts of interest.

## Data Availability

The data that support the findings of this study are available on request from the corresponding author. The data are not publicly available due to privacy or ethical restrictions.
